# Semi-field and indoor setups to study malaria mosquito swarming behavior

**DOI:** 10.1186/s13071-019-3688-0

**Published:** 2019-09-11

**Authors:** Abdoulaye Niang, Charles Nignan, B. Serge Poda, Simon P. Sawadogo, K. Roch Dabiré, Olivier Gnankiné, Frédéric Tripet, Olivier Roux, Abdoulaye Diabaté

**Affiliations:** 1 Institut de Recherche en Sciences de la Santé (IRSS), Bobo-Dioulasso, Burkina Faso; 2 Laboratoire d’Entomologie Fondamentale et Appliquée, Unité de Formation et de Recherche en Sciences de la Vie et de la Terre (UFR-SVT), Université Ouaga I Pr. Joseph KI-ZERBO, Ouagadougou, Burkina Faso; 3 MIVEGEC, IRD, CNRS, University of Montpellier, Montpellier, France; 4 Centre for Applied Entomology and Parasitology, School of Life Sciences, Keele University, Staffordshire, UK

**Keywords:** *Anopheles*, Burkina Faso, Mating behavior, Mosquito ecology research facility, Swarming room

## Abstract

**Background:**

The recent resurgence of interest in sterile insect techniques to control vector mosquitoes has renewed interest in novel methods for observing mating behavior. Malarial vectors of the *Anopheles gambiae* complex are known to mate in swarms at specific locations at dawn and dusk. Most knowledge of mosquito swarming behavior is derived from field observations and a few experimental studies designed to assess critical parameters that affect mosquito swarming. However, such studies are difficult to implement in the field because of uncontrollable environmental factors and mosquito conditions. Here, we present two experimental setups specifically designed to analyze mosquito swarming behavior and provide evidence that swarming behavior of mosquitoes can be generated and accurately assessed under both semi-field and laboratory conditions.

**Methods:**

The Mosquito Ecology Research Facility setup is a semi-field enclosure made of 12 compartments (10.0 × 6.0 × 4.5 m L × W × H each) exposed to ambient meteorological and lighting conditions. The laboratory setup consists of a windowless room (5.1 × 4.7 × 3.0 m) in which both environmental and mosquito conditions can be controlled. In the two setups, 300 3–6-days-old *An. coluzzii* virgin males were released and some swarm characteristics were recorded such as the time at which the swarm started, the number of mosquitoes in the swarm and the height. Climatic conditions in the semi-field setup were also recorded.

**Results:**

In both setups, *An. coluzzii* males displayed stereotyped and consistent swarming behavior day after day; males gradually gather into a swarm over a ground marker at sunset, flying in loops in relation to specific visual features on the ground. Although semi-field climatic conditions were slightly different from outdoors conditions, they did not impede swarming behavior and swarm characteristics were similar to those observed in the field.

**Conclusions:**

Swarm characteristics and their consistency across days provide evidences that these facilities can be used confidently to study swarming behavior. These facilities come to complement existing semi-field setups and pave the way for new experimental studies which will enhance our understanding of mating behavior but also mosquito ecology and evolution, a prerequisite for application of genetic approaches to malaria control.

## Background

Malaria transmission is vectored by the bite of an infected *Anopheles* female mosquito. Consequently, most studies carried out during the last decades focused on female biting behavior. Other behaviors in their life-cycle, such as oviposition, resting, sugar-feeding and mating have been overlooked [1, 2], and yet, they are keystones in the mosquito life-cycle, acting as bottlenecks which, if females fail to accomplish, will compromise their fitness and that of their progeny.

Mating behavior is responsible for reproductive isolation and species diversification, which have important implications for vector control strategies targeting the *Anopheles gambiae* complex [3–5]. Understanding the processes involved in species-specific mating behavior is crucial because it can pave the way to design novel tools to attract, trap and kill males and females [6], to improve the mating competitiveness of mass-produced males used for release programmes and to target residual transmission by trapping young virgin females.

In the *An. gambiae* complex, mating occurs in swarms formed by a few to thousands males at sunset that last for 20–30 minutes [7]. Males gather over conspicuous landmarks, such as an area of dark/light contrast on the ground (aka ‘swarm markers’) or over objects that interrupt the regularity of a visually smooth landscape. Females join the swarm, find a mate and leave the swarm in copula (see [8] for review). Interestingly, males systematically use the same swarm sites over time and swarms are consistently found at the same locations year after year [3, 9]. Moreover, hetero-specific swarms are rare [10]. Overall, this suggests that species-specific cues are used by males to form swarms. This mating behavior is consistent across malaria vector species prevalent in open-field habitats (*An. gambiae* (*s.s.*), *An. coluzzii*, *An. arabiensis*, *An. melas*, *An. funestus*) and across geographic regions in Africa, including Burkina Faso, Mali, Benin, Sudan, Cameroon, Gambia, Sao Tome et Principe, Mozambique, Tanzania [4, 11–18].

Current knowledge of swarming behavior comes predominantly from field observations. Some swarm characteristics such as size, height, number of couples, both starting and ending times have been recorded [3, 4, 7, 9, 19, 20]. Although field experiments have been attempted by manipulating swarm markers [8, 14], it is difficult to control for environmental and mosquito parameters that lead to swarm formation in the field, which makes the interpretation of results questionable and restricts progress in our understanding of mating behavior. With this in mind, setups have been built and have shown that *Anopheles* species were able to produce swarms, mate and reproduce in semi-field setups [21–24]; however, tools specially designed for experimental swarming behavior studies are needed. Such tools need to provide conditions allowing to trigger swarming behavior but also should allow to control for visual parameters used by mosquitoes to locate their swarms (i.e. visual markers in an empty space). Here, we describe and provide proof of concept for two experimental setups specifically designed to generate, observe and manipulate mosquito swarms; one in semi-field conditions and the other in the laboratory.

## Methods

### Mosquitoes

Experiments were conducted with F1 mosquitoes obtained from wild females collected in Bama (11° 24′ 14″ N, 4° 24′ 42″ W), a village located 30 km north of Bobo-Dioulasso, Burkina Faso. Indoor-resting gravid females belonging to the *Anopheles* genus were collected using mouth aspirators and transferred to the insectarium. Females were placed individually in oviposition cups containing tap water. After oviposition, females were identified to species by routine PCR-RFLP [25]. Newly hatched larvae from females identified as *An. coluzzii* were pooled and fed Tetramin® Baby Fish Food (Tetrawerke, Melle, Germany) *ad libitum*. At emergence, adults were sexed and maintained separately in 30 × 30 × 30 cm cages and provided with 5% glucose solution *ad libitum*.

### Mosquito ecology research facility (MERF) design:- semi-field conditions

**Fig. 1:**
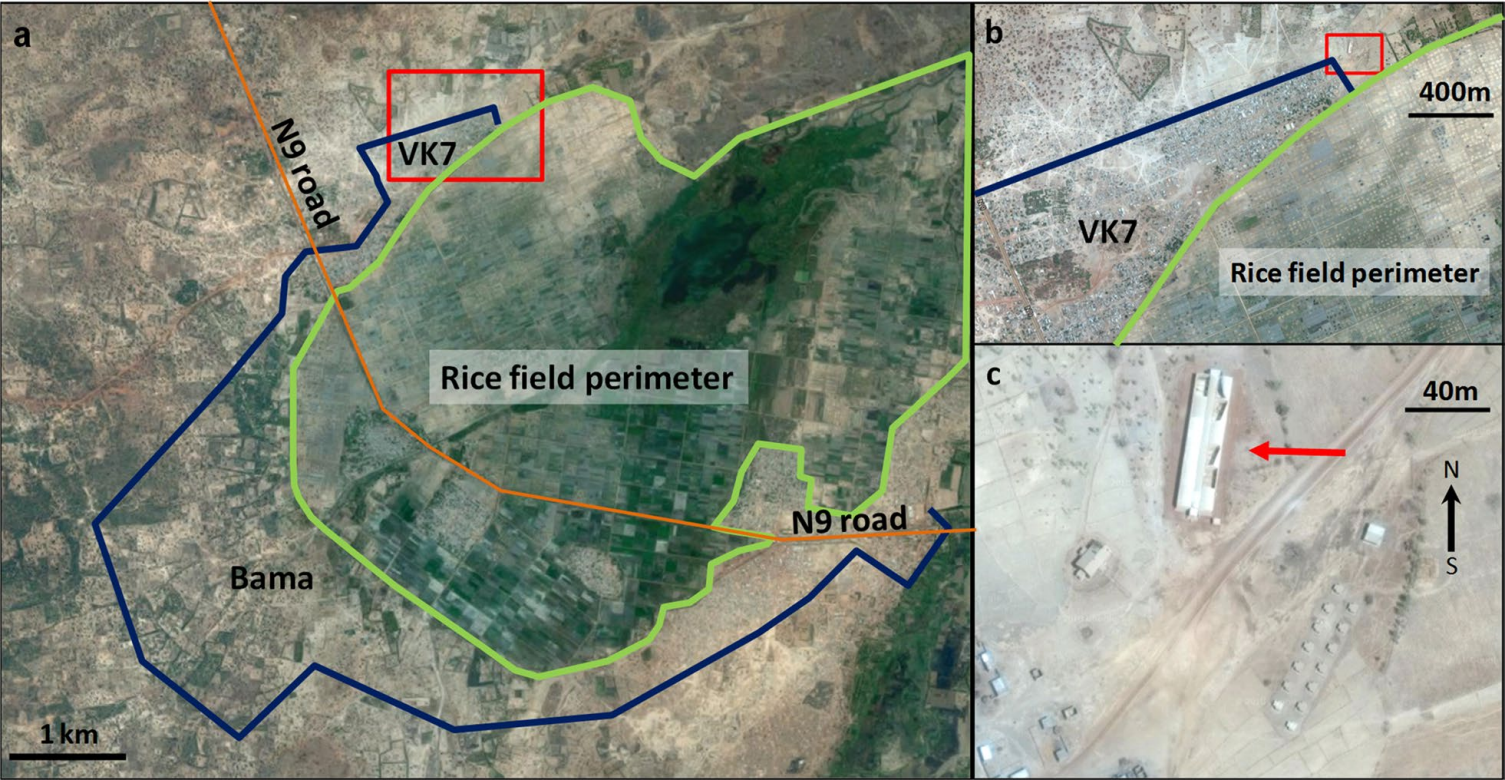
Aerial view of Bama area where the field station of the Institut de Recherche en Sciences de la Santé is settled. **a** Global view with the rice field perimeter (green line), the village limits (blue line) and the district VK7 in which the semi-field setup is located (red square). **b** Closer view of VK7 area. **c** View of the field station at VK7 (red arrow: MERF)

The Mosquito Ecology Research Facility (MERF, hereafter) is a semi-field research facility located in Bama (Fig. 1a, b). Bama is a rice-growing area covering 1200 ha and surrounded by humid savannah where the rainy season extends from June to October and the dry season from November to May. The irrigation system and rice fields provide year-round mosquito breeding sites. Malaria transmission intensity within this area is high, with up to 200 bites/person/night during the rainy season [26]. This area has been subjected to extensive studies of swarming behavior, notably in *An. gambiae* (*s.s.*) and *An. coluzzii* since 2003 [7, 8], providing a good background for comparison between field and semi-field studies.

**Fig. 2:**
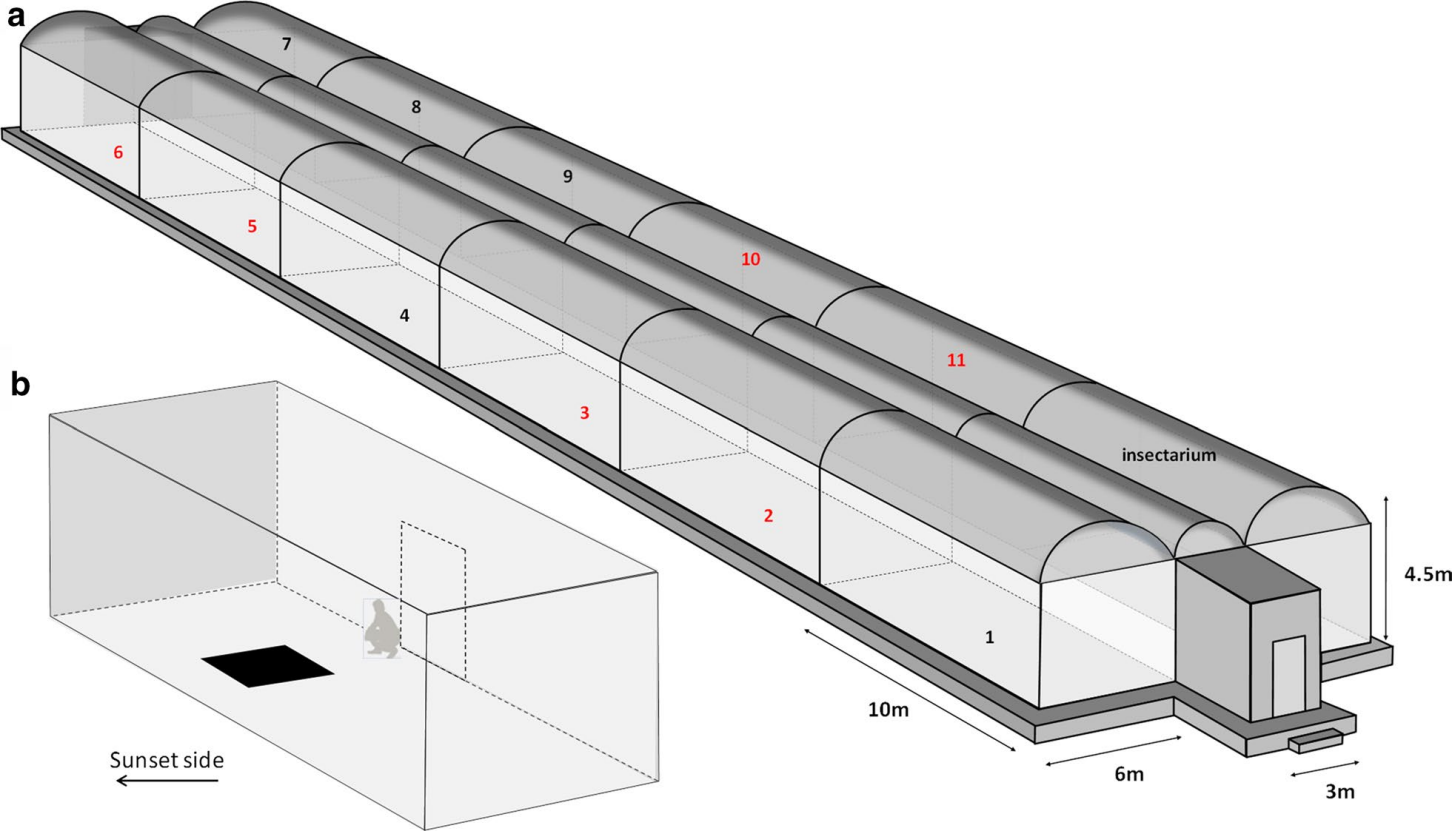
**a** Schematic view of the MERF. Compartments are numbered from 1 to 11. Red numbers indicate the compartments used in the experiment. Compartment 1–6 are facing the sunset side. **b** Compartment with experimental design (black square is the 1.5 × 1.5 m ground marker)

The MERF was built in 2013 and was inspired and adapted from the existing semi-field “Malaria Spheres” in East Africa at Mbita Point Research & Training Centre (ICIPE, Kenya) [23] and at the Ifakara Health Institute in Tanzania [22]. The MERF is oriented approximately along a north-south axis (Fig. 1c) to provide homogeneous lighting from the sky along its long side at sunset. The floor area is ~900 m^2^ and raised ~0.5 m above ground level to avoid flooding during the rainy season. The interior consists of two rows of six compartments, 10.0 × 6.0 × 4.5 m each (L × W × H), with a total working floor-area of 60 m^2^ and a volume of 257 m^3^. The two rows of compartments are separated by a 3-m long corridor down the axis of the enclosure (Fig. 2a, b). Each end of this corridor opens into an antechamber (3.0 × 2.5 × 2.4 m; L × W × H) made of concrete, which provides secure access to the outdoors. The MERF is made of an iron framework fixed on a concrete floor. Walls are made of polyester netting (Polytex), with 346 holes/inch^2^ allowing airflow through the compartment ensuring climatic conditions similar to the surrounding ambient conditions. The roof consists of a 200 µm thin transparent polyane thermic film (Celloclim® 4S LC), allowing optimal light diffusion and limiting high temperature peaks. Each compartment is equipped with access to electricity and tap water. The MERF is connected to the national electric system and a generator can take over in case of a power cut. One compartment is used as a semi-field insectarium, equipped with air-conditioning that maintains the ambient temperature at ~25–30 °C, with natural light levels and relative humidity.

### Swarming room design: laboratory conditions

**Fig. 3:**
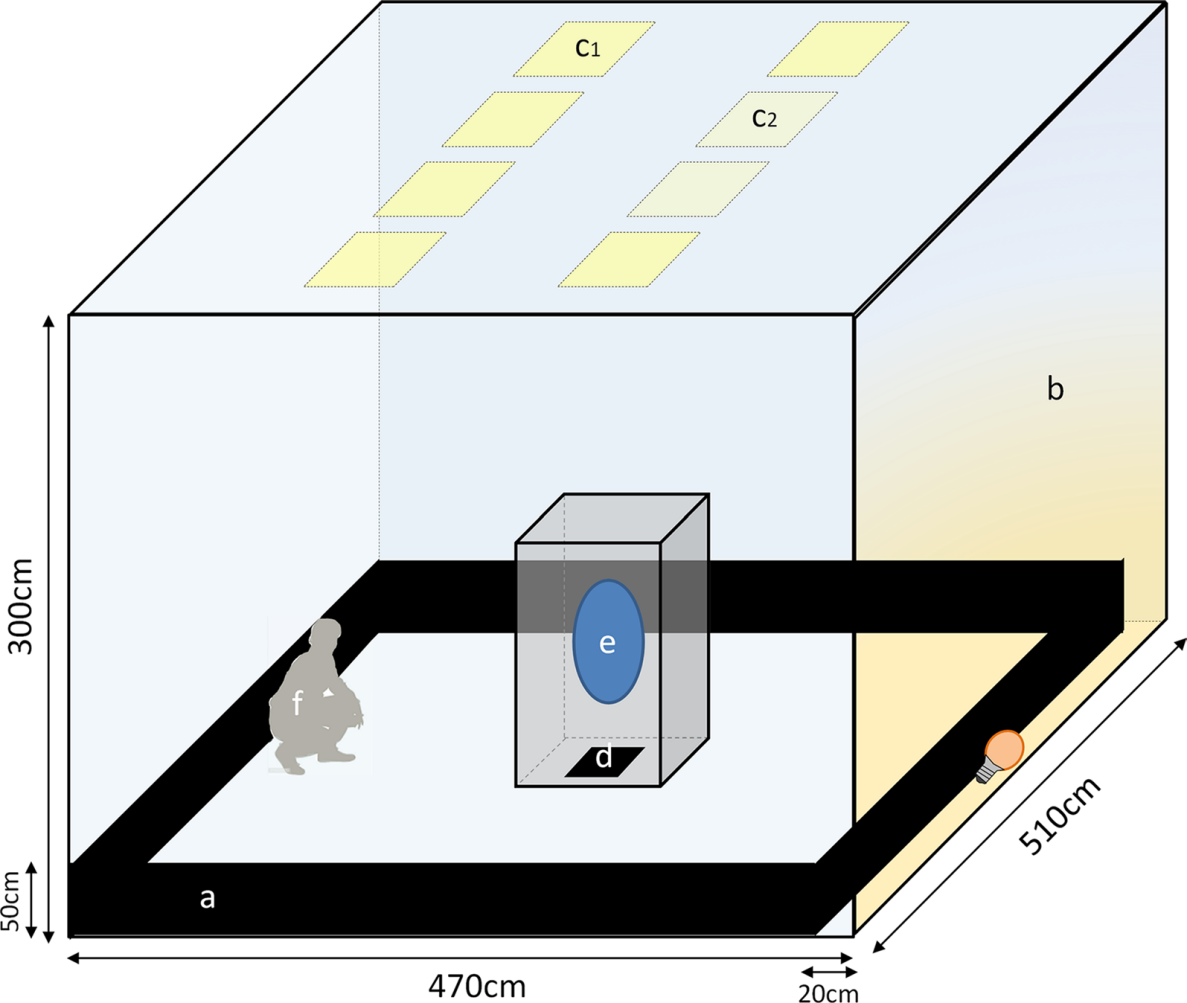
Schematic view of the swarming room with stimuli necessary to stimulate swarming behavior: a black horizon; b bright wall with an incandescent bulb behind the black horizon; c_1_ and c_2_ programmed ceiling light (LED panels) to simulate sunset; d ground marker; e Swarm into a cage; f observer looking at the swarm with the bright wall as background

The swarming room is on the campus of IRSS in Bobo-Dioulasso and was designed and constructed to stimulate indoor swarming behavior in fully controlled environmental conditions to minimize confounding effects between treatments and environmental factors. The design was largely inspired from the previous work of Facchinelli et al. [27] and others [14, 28, 29], but kept as simple as possible to make the room adaptable for different experimental topics and designs (Fig. 3).

The swarming room is 5.1 × 4.7 × 3.0 m (L × W × H) and ~24 m^2^ working floor area and 72 m^3^ volume, with white walls, ceiling and floor and no windows or natural light. It is equipped with an air conditioner for temperature control and a humidifier (Condair 505 Defensor) to control relative humidity. According to Facchinelli et al. [27], four visual stimuli are necessary to obtain consistent swarms, which are reproduced here (Fig. 3): (i) ceiling lights that can be controlled to simulate dusk (Fig. 3; c1 and c2). These ceiling lights consist of 8 ultra-slim LED panels (60 × 60 cm, 43 W, 4000 K) fixed on the ceiling in two lines and controlled by a Sunlite Touch-sensitive Intelligent Control Keypad (STICK-KE1, Nicolaudie, Paris, France). Fading is programmed with ESA Pro software (Computerized Lighting Controller, Nicolaudie); (ii) a black artificial horizon which is made of 50 cm high black cloth placed all around the room at the bottom of the walls (Fig. 3a); (iii) a “bright horizon” to simulate twilight consists of a 40 W incandescent bulb (2500 K) located on the floor between a wall and the black horizon; (iv) and a conspicuous visual marker consisting of black cloth (60 × 60 cm) is placed in the center of the inner cage to serve as the stationary marker (Fig. 3d), over which mosquitoes swarmed (Fig. 3e).

### Experimental design

#### MERF experiments

About 300 3–5-days-old *An. coluzzii* virgin males were transferred to the MERF 2 h before sunset. Six compartments were used simultaneously. In each compartment, a black cloth (1.5 × 1.5 m) was randomly placed on the floor as a swarm marker (Fig. 2b) and the 300 males were released 30 min before sunset. Observations started as soon as the first males initiated the swarm and this time point was recorded (hereafter, ‘swarm start time’). Fifteen minutes later, the height of the swarm nucleus (defined as the height of the highest mosquito density into the swarm) and the numbers of mosquitoes in the swarm were estimated by eye. Then, mosquitoes were collected with a net and counted. Measurements were made by one observer per compartment located ~2 m from the swarm (Fig. 2b). Six replicates were run with a complete rotation of the six observers.

Temperature and relative humidity inside and outside the MERF were recorded at 3 time points around swarming time (18:00 h, 19:00 h and 20:00 h); MERF compartment number 3 was fitted with a MSR®145 datalogger, and outside the MERF a weather station Vantage Pro2 (Weatherlink, Davis Instruments, USA) was located ~90 m away.

#### Swarming room experiments

About 300 4–6-days-old *An. coluzzii* virgin males were released into a vertical cage (70 × 70 × 150 cm; L × W × H; Fig. 3) within the main room at least 30 min before the programmed sunset started to allow the mosquitoes to acclimatize. The cage frame was painted white and covered with white net and placed in the middle of the room. A 60 × 60 cm black marker was located on the floor in the middle of the cage (Fig. 3d). To trigger swarming behavior, the ceiling lights were dimmed from 100% of their power to 0% over 30 min. To avoid a too sharp change in light intensity at the end dusk fading, 6 of the 8 LED panels (Fig. 3, C_1_) were programmed to turn off first. The last two panels (Fig. 3, C_2_) were programmed to turn off 5 min later. During the whole process, the horizon light stayed “on” even after the ceiling lights were turned off (see Additional file 1: Figure S1). This arrangement of lights provided a clear background which allowed easy observation of mosquito flight by eye (Fig. 3e shows swarming area). Mosquitoes were observed from 10 min before the ceiling light extinction until the end of swarming behavior period. As previously, the height of the swarm nucleus and the number of swarming mosquitoes were estimated by eye 15 min after mosquitoes started to swarm. The height was evaluated thanks to a graduated adhesive tape stuck on the cage frame. Twelve replicates were performed.

### Statistical analysis

All analyses were performed using R (version 3.4.0). The mean temperature and relative humidity as a function of time (18:00 h, 19:00 h or 20:00 h) and location (inside or outside the MERF) were analyzed separately with Generalized Linear-Mixed Model (GLMM, *lme4* package). Time, location and their interactions were considered fixed effects. Days were considered as random effects.

In the MERF, swarming rate (i.e. the proportion of males joining the swarm) was analyzed with a binomial GLMM and using the ‘number of males collected in the swarms at the end of each swarm observation’ which were not statistically different from the ‘estimated number of swarming males’ (see “Results” section). Height of the nucleus and time at which mosquitoes started to swarm were analyzed separately using a gaussian GLMM. For all models, compartments were considered as fixed effects. Days and observers were considered random effects.

For model selection, we used the stepwise removal of terms, followed by likelihood ratio tests. Term removals that significantly reduced explanatory power (*P* < 0.05) were retained in the minimal adequate model [30].

## Results

**Fig. 4:**
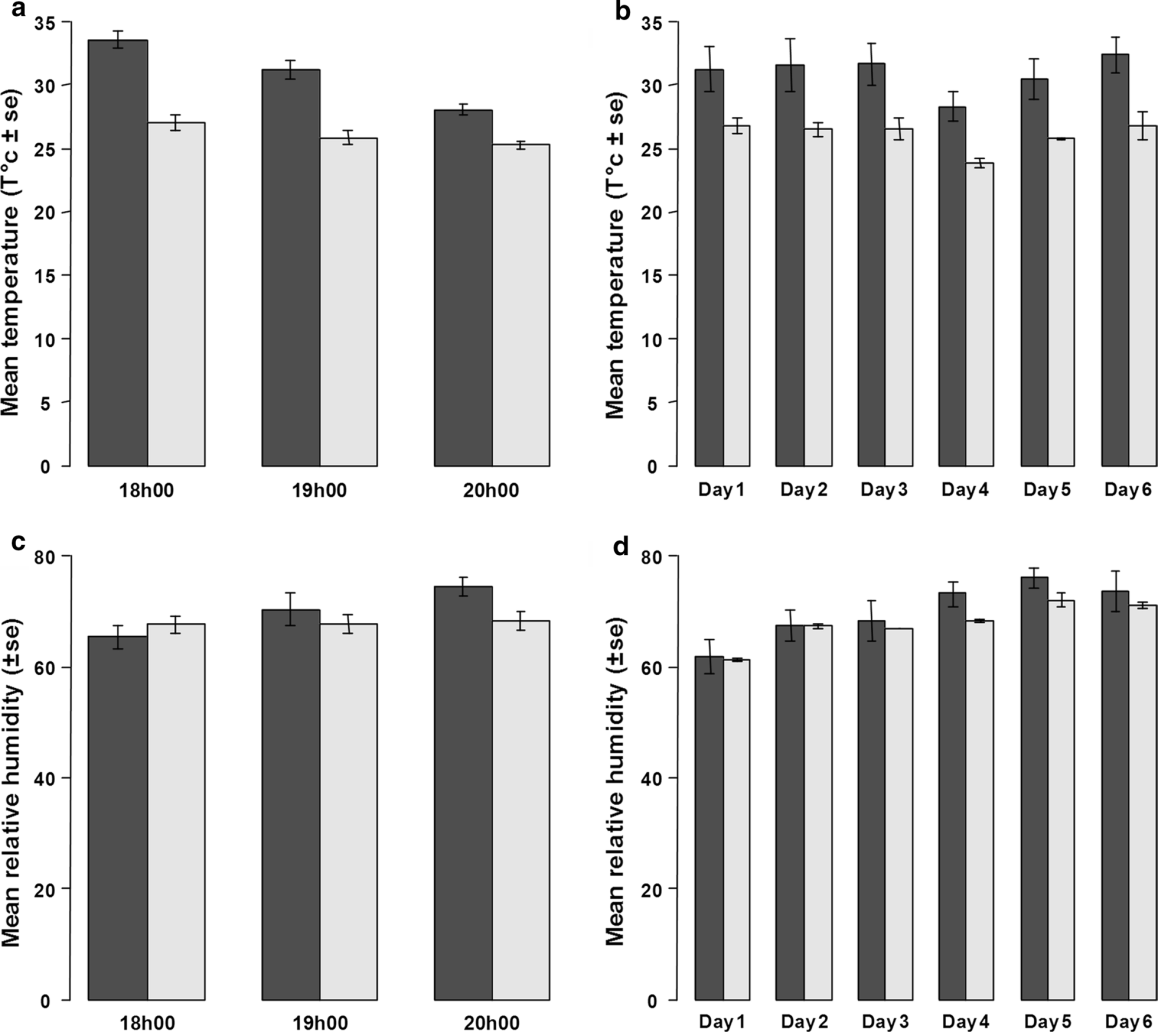
Climatic conditions during the semi-filed experiment into (dark grey) and out of (light grey) the MERF. Mean temperatures (± SE) around swarming time (**a**) and across days (**b**). Mean relative humidity (± SE) around swarming time **c** and across days **d**

Mean temperatures (± standard error, SE) at swarming time in the MERF were significantly higher than outside (30.9 ± 0.6 °C *vs* 26.0 ± 0.3 °C, respectively; *χ*^2^= 249, *df *= 1, *P *< 0.001). Temperatures decreased significantly with time (*χ*^2^= 177, *df *= 2, *P *< 0.001). However, the temperature dropped faster inside the MERF than outside (time-location interaction: *χ*^2^= 40.8, *df *= 2, *P* < 0.001; Fig. 4a; see Additional file 1: Table S1). These differences were consistent across days (Fig. 4b).

Mean relative humidity (± SE) in the MERF was not significantly different from outside (67.8 ± 0.8% *vs* 70.0 ± 1.5% respectively; *χ*^2^= 3.7, *df *= 1, *P *= 0.06; Fig. 4c, d). However, an increase in relative humidity with time was recorded inside the MERF, but not outside (time-location interaction: *χ*^2^= 21.8, *df *= 2, *P* < 0.001; Fig. 4c; see Additional file 1: Table S1).

**Fig. 5:**
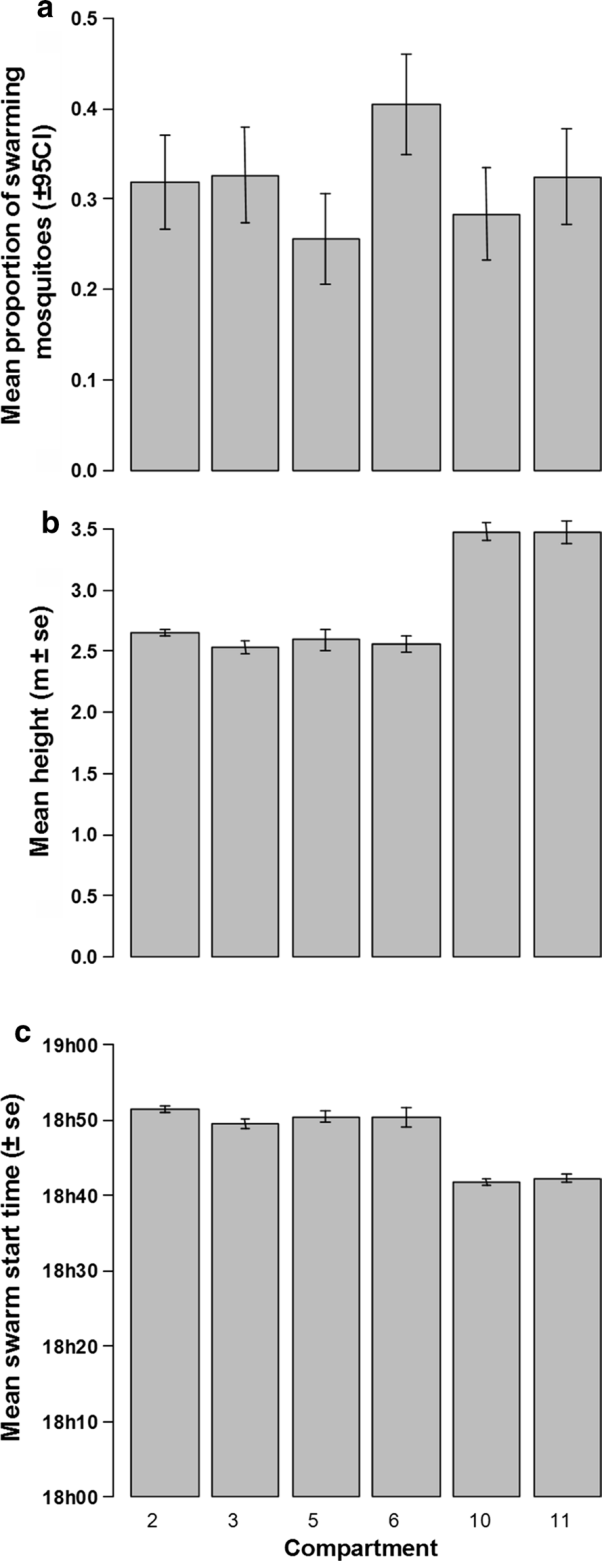
Swarm characteristics into each compartment of the MERF. **a** Mean proportion of swarming mosquitoes based on captured mosquitoes. **b** Mean height of the swarm nucleus (higher density of mosquitoes into the swarm). **c** Mean time at which the first mosquitoes started to swarm over the ground marker

MERF experiments consisted of a total of 36 observed swarms. The mean estimated number of males in the swarms was not statistically different from the mean number of males collected in the swarms at the end of each observation period (94.7 ± 5.2 *vs* 95.0 ± 5.6; ~31% of released mosquitoes; paired t-test: *t*_(35)_= −  0.24, *P *= 0.81). No significant difference in swarming rate was observed between compartments (*χ*^2^= 9.8, *df *= 5, *P *= 0.08; Fig. 5a; see Additional file 1: Table S2). The mean estimated height (± SE) at which males were flying was 2.8 ± 0.1 m. However, they flew higher in compartments 10 and 11 (3.47 ± 0.05 m) compared to the other compartments (2.58 ± 0.03 m) (compartment effect: *χ*^2^= 78, *df *= 5, *P* < 0.001, Tukeyʼs *post-hoc* tests *P* < 0.001; Fig. 5b). Similarly, mosquitoes formed swarms ~8 min sooner in compartments 10 and 11 compared to the other compartments (18:42 h *vs* 18:50 h; *χ*^2^= 69, *df *= 5, *P* < 0.001, Tukeyʼs *post-hoc* tests *P* < 0.001, Fig. 5c).

In the swarming room experiments, the visual stimuli consistently triggered swarming behavior for the 12 assays; ~5 min before the ceiling lights went off, mosquitoes started to fly randomly through the cage. Those that were not flying yet had their antennae erect. About 2 min after the ceiling lights went off, 2–3 mosquitoes started to fly over the marker in loops, as expected. Their number increased rapidly within the first 2 min, with an estimated mean number (± SE) of 74.0 ± 2.4 mosquitoes (*~*23% of released mosquitoes) flying at 1.1 ± 0.1 m over the marker. The mean number of mosquitoes in the swarms was consistent during the first 30 min, then the number of mosquitoes decreased. Nevertheless, some mosquitoes continued to swarm for ~1 h after the ceiling lights went off. If the horizon light went off at any time, swarming behavior ceased.

## Discussion

We present here setups designed to study mosquito swarming and mating behavior in semi-field and in laboratory conditions and provide data complementary to that of field studies. Such setups allow the control of critical parameters, such as mosquito physiology, the physical environment (e.g. visual markers and light intensity) and/or climatic conditions that might affect mosquito swarming.

Climatic conditions in our semi-field setup were different from the outdoor ambient conditions. Nevertheless, temperature and relative humidity fluctuations were similar across days and during experiments. Inside temperatures were significantly higher than outside temperatures, but without impeding swarming behavior. Temperatures exceeding 30 °C for several hours each day are supposed to be lethal for adult mosquitoes [22]. However, the purpose of the MERF is not for longitudinal studies but for swarming behavior studies only, which take place for 20–30 min in the evening when temperatures drop. Moreover, temperatures recorded in the MERF are not ecologically irrelevant in Burkina Faso as temperatures above 30 °C at sunset were recorded all around the year with the weather station near the MERF (Additional file 1: Figure S2). Our observations show that mosquitoes swarm in the MERF without obvious differences in behavior to natural swarms observed outside, indicating that the environmental conditions in the MERF are suitable for swarming studies. Moreover, released mosquitoes continued to fly randomly in the compartments several hours after the typical swarming time ended, indicating that indoor climatic conditions during the night do not affect mosquito survivorship and activity patterns. During the day, temperatures increase and the absence of water and carbohydrate resources allowed us to purge the compartment of mosquitoes released the day before, which enables a new cohort to be tested each day. Nevertheless, longitudinal studies are possible if refuges (wet clay pots) and carbohydrate sources (plants) are introduced into the compartments (data not shown).

Swarming behavior was highly reproducible; swarms were composed of relatively constant numbers of swarming mosquitoes and at a consistent height across days. Estimated numbers of swarming mosquitoes and the numbers of collected swarming mosquitoes in the MERF were also very similar, indicating that environmental conditions enabled us to obtain valuable swarm observations. Moreover, swarm characteristics recorded in the MERF were consistent with those recorded in the field in previous studies at the same locality with swarm size ranging from about 10 to more than 800 males in august [3, 4, 7] and flying at a mean height of 2 m (range from 0.5 to 5 m) [4, 9]. However, it is difficult to compare such characteristics between semi-field and field as the semi-field population is finite and because the marker size could play a role in swarm size [31]. As in Achinko et al. [21], time at which mosquitoes started to swarm in the MERF was also consistent across days and was not different than time recorded in the field at the same period by Sawadogo et al. [19] (18:43 h in August).

Despite the consistency of swarm characteristics across days, differences were detected between some compartments. Indeed, mosquitoes flew higher and formed swarms a bit earlier in compartments 10 and 11 compared to other compartments. As these two compartments are at the opposite side of the MERF to sunset, they were exposed to a lower light intensity than the other compartments; light intensity is known to be a critical parameter in triggering swarm formation [19] and could be responsible for these differences in swarm characteristics.

Swarming behavior in the swarming room was also highly reproducible, but the number of estimated swarming mosquitoes and the swarm height above the ground were different than that observed in the MERF, swarms in the swarming room were smaller and flew lower than in the MERF. It is worth noting that in the swarming room, mosquitoes were contained in a cage and that the swarming room dimensions were different from those of the MERF compartments. Importantly, however, mosquitoes reacted similarly to the same stimuli in both the MERF and the swarming room. Moreover, mosquito behavior was very similar to that described by Charlwood & Jones [14], with mosquitoes having their antennae erect and flying in loops over the ground marker after the light was dimmed.

Studying swarms in the laboratory provides several benefits. Working in a dark room with a programmable sunset makes it possible to produce swarms several times a day. However, mosquitoes need to be kept under specific dark:light regimes, to control for the circadian activity rhythms of mosquitoes [32], to ensure that experiments are conducted at the scotophase when the mosquito species is expected to swarm. Moreover, swarming behavior can last longer in the laboratory setup than under semi-field conditions if the lighting is managed correctly. For example, as long as the bright horizon light was on, some mosquitoes swarmed. This behavior is not unnatural, as Charlwood & Jones (1980) observed similar behavior in natural *An. melas* swarm when a full moon was present. The swarming room is also fully adjustable, and can accommodate a wide range of cage sizes or can be used even without cages. All types of visual markers can be used and light cycles can be manipulated for longitudinal studies.

## Conclusions

The MERF and the swarming room setups are highly efficient to generate and observe mosquito swarms in a consistent and controlled manner for accurate behavioral experimentations. These facilities come to complement existing semi-field systems with more natural conditions (shelters, plants and breeding sites) in which it was proved that *Anopheles* species are able to complete their life-cycle [21–24]. All together, they can help to shed light on mosquito mating behavior and be used to further improve our knowledge of both mosquito mating and evolutionary ecology which can lead to improved vector surveillance and control approaches.

## Supplementary information

**Additional file 1: Table S1.** Effects of time of the day (18 h, 19 h or 20 h), location (inside or outside the MERF) and their interaction on mean temperature and humidity. **Table S2.** Effects of MERF compartments on swarming rate, the height of swarm nucleus and the time at which the swarms started. **Figure S1.** Light stimuli used into the swarming room. **a** Ceiling light program with LED panels dimmed from 100% to 0% with the two C_2_ panels turning off 5 minutes after C_1_ panels. **b** Incandescent bulb light illuminating the bright wall during all the experiment. **Figure S2:** Monthly temperatures outside the MERF and in the MERF during experiments.

## Data Availability

Data supporting the conclusions of this article are included within the article and its additional file. The raw datasets are available from the corresponding author upon reasonable request.

## References

[1] Ferguson HM, Dornhaus A, Beeche A, Borgemeister C, Gottlieb M, Mulla MS (2010). Ecology: a prerequisite for malaria elimination and eradication. PLoS Med..

[2] Takken W, Costantini C, Dolo G, Hassanali A, Sagnon N, Osir E, Knols BGJ, Louis C (2006). Mosquito mating behaviour. Bridging laboratory and field research for genetic control of disease vectors.

[3] Diabaté A, Dao A, Yaro AS, Adamou A, Gonzalez R, Manoukis NC (2009). Spatial swarm segregation and reproductive isolation between the molecular forms of *Anopheles gambiae*. Proc R Soc B..

[4] Diabaté A, Yaro AS, Dao A, Diallo M, Huestis DL, Lehmann T (2011). Spatial distribution and male mating success of *Anopheles gambiae* swarms. BMC Evol Biol..

[5] Tripet F, Thiemann T, Lanzaro GC (2005). Effect of seminal fluids in mating between M and S forms of *Anopheles gambiae*. J Med Entomol..

[6] Sawadogo SP, Niang A, Bilgo E, Millogo A, Maïga H, Dabire RK (2017). Targeting male mosquito swarms to control malaria vector density. PLoS One..

[7] Diabaté A, Baldet T, Brengues C, Kengne P, Dabiré KR, Simard F (2003). Natural swarming behaviour of the molecular M form of *Anopheles gambiae*. Trans R Soc Trop Med Hyg..

[8] Diabaté A, Tripet F (2015). Targeting male mosquito mating behaviour for malaria control. Parasit Vectors.

[9] Sawadogo PS, Namountougou M, Toé KH, Rouamba J, Maïga H, Ouédraogo KR (2014). Swarming behaviour in natural populations of *Anopheles gambiae* and *An. coluzzii*: Review of 4 years survey in rural areas of sympatry, Burkina Faso (West Africa). Acta Trop..

[10] della Torre A, Tu Z, Petrarca V (2005). On the distribution and genetic differentiation of *Anopheles gambiae s.s.* molecular forms. Insect Bioch Mol Biol..

[11] Diabaté A, Dabiré RK, Kengne P, Brengues C, Baldet T, Ouari A (2006). Mixed swarms of the molecular M and S forms of *Anopheles gambiae* (Diptera: Culicidae) in sympatric area from Burkina Faso. J Med Entomol..

[12] Assogba BS, Djogbénou L, Saïzonou J, Diabaté A, Dabiré RK, Gilles J (2014). Characterization of swarming and mating behaviour between *Anopheles coluzzii* and *Anopheles melas* in a sympatry area of Benin. Acta Trop..

[13] Charlwood JD, Jones MDR (1979). Mating behaviour in the mosquito, *Anopheles gambiae s.l.* I. Close range and contact behaviour. Physiol Entomol..

[14] Charlwood JD, Jones MDR (1980). Mating in the mosquito, *Anopheles gambiae s.l.* II. Swarming behavior. Physiol Entomol..

[15] Charlwood JD, Pinto J, Sousa CA, Madsen H, Ferreira C, do Rosario VE (2002). The swarming and mating behaviour of *Anopheles gambiae s.s.* (Diptera: Culicidae) from Sao Tome Island. J Vector Ecol..

[16] Charlwood JD, Thompson R, Madsen H (2003). Observations on the swarming and mating behaviour of *Anopheles funestus* from southern Mozambique. Malar J..

[17] Marchand RP (1984). Field observations on swarming and mating in *Anopheles gambiae* mosquitoes in Tanzania. Neth J Zool..

[18] Kaindoa EW, Ngowo HS, Limwagu AJ, Tchouakui M, Hape E, Abbasi S (2019). Swarms of the malaria vector *Anopheles funestus* in Tanzania. Malar J..

[19] Sawadogo SP, Costantini C, Pennetier C, Diabaté A, Gibson G, Dabiré RK (2013). Differences in timing of mating swarms in sympatric populations of *Anopheles coluzzii* and *Anopheles gambiae s.s.* (formerly *An. gambiae* M and S molecular forms) in Burkina Faso. West Africa. Parasit Vectors..

[20] Sawadogo SP, Diabate A, Toe HK, Sanon A, Lefevre T, Baldet T (2013). Effects of age and size on *Anopheles gambiae s.s.* male mosquito mating success. J Med Entomol..

[21] Achinko D, Thailayil J, Paton D, Mireji PO, Talesa V, Masiga D (2016). Swarming and mating activity of *Anopheles gambiae* mosquitoes in semi-field enclosures. Med Vet Entomol..

[22] Ferguson HM, Ng’habi KR, Walder T, Kadungula D, Moore SJ, Lyimo I (2008). Establishment of a large semi-field system for experimental study of African malaria vector ecology and control in Tanzania. Malar J..

[23] Knols BG, Njiru BN, Mathenge EM, Mukabana WR, Beier JC, Killeen GF (2002). MalariaSphere: a greenhouse-enclosed simulation of a natural *Anopheles gambiae* (Diptera: Culicidae) ecosystem in western Kenya. Malar J..

[24] Munhenga G, Brooke BD, Chirwa TF, Hunt RH, Coetzee M, Govender D (2011). Evaluating the potential of the sterile insect technique for malaria control: relative fitness and mating compatibility between laboratory colonized and a wild population of *Anopheles arabiensis* from the Kruger National Park, South Africa. Parasit Vectors..

[25] Fanello C, Santolamazza F, della Torre A (2002). Simultaneous identification of species and molecular forms of the *Anopheles gambiae* complex by PCR-RFLP. Med Vet Entomol..

[26] Baldet T, Diabaté A, Guiguemde TR (2003). Malaria transmission in 1999 in the rice field area of the Kou Valley (Bama), (Burkina Faso). Santé..

[27] Facchinelli L, Valerio L, Lees R, Oliva C, Persampieri T, Collins C (2015). Stimulating *Anopheles gambiae* swarms in the laboratory: application for behavioural and fitness studies. Malar J..

[28] Marchand RP (1985). A new cage for observing mating behavior of wild *Anopheles gambiae* in the laboratory. J Am Mosq Ctrl Assoc..

[29] Gibson G (1985). Swarming behaviour of the mosquito *Culex pipiens quinquefasciatus*: a quantitative analysis. Physiol Entomol..

[30] Crawley MJ (2007). The R book.

[31] Downes JA (1969). The swarming and mating flight of Diptera. Ann Rev Entomol..

[32] Jones MDR, Gubbins PS (1978). Changes in the circadian flight activity of the mosquito *Anopheles gambiae* in relation to insemination, feeding and oviposition. Physiol Entomol..

