# Supplementary material for: Semi-field and indoor setups to study malaria mosquito swarming behavior
Source: Parasit Vectors. 2019 Sep 11;12:446. doi: 10.1186/s13071-019-3688-0 (PMC6737701; doi:10.1186/s13071-019-3688-0)
Supplement: Supplementary file 1 — Additional file 1: Table S1. Effects of time of the day (18 h, 19 h or 20 h), location (inside or outside the MERF) and their interaction on mean temperature and humidity. Table S2. Effects of MERF compartments on swarming rate, the height of swarm nucleus and the time at which the swarms started. Figure S1. Light stimuli used into the swarming room. a Ceiling light program with LED panels dimmed from 100% to 0% with the two C2 panels turning off 5 minutes after C1 panels. b Incandescent bulb light illuminating the bright wall during all the experiment. Figure S2: Monthly temperatures outside the MERF and in the MERF during experiments. [file 13071_2019_3688_MOESM1_ESM.docx]

**Additional file 1**

**Semi-field and indoor setups to study malaria mosquito swarming behavior**

**Abdoulaye Niang^1^, Charles Nignan^1,2^, B. Serge Poda^1,2,3^, Simon P. Sawadogo^1^, K. Roch Dabiré^1^, Olivier Gnankiné^2^, Frédéric Tripet^4^, Olivier Roux^1,3†^ and Abdoulaye Diabaté^1†*^**

^1^Institut de Recherche en Sciences de la Santé (IRSS), Bobo-Dioulasso, Burkina Faso.

^2^Laboratoire d’Entomologie Fondamentale et Appliquée, Unité de Formation et de Recherche en Sciences de la Vie et de la Terre (UFR-SVT), Université Ouaga I Pr. Joseph KI-ZERBO, Ouagadougou, Burkina Faso.

^3^MIVEGEC, IRD, CNRS, University of Montpellier, Montpellier, France.

^4^Centre for Applied Entomology and Parasitology, School of Life Sciences, Keele University, Staffordshire, UK.

*correspondence: npiediab@gmail.com

† Olivier Roux and Abdoulaye Diabaté contributed equally to this work

**Additional file 1: Table S1: Effects of time of the day (18:00h, 19:00h or 20:00h), location (inside or outside the MERF) and their interaction on mean temperature and humidity.**

| Mean temperature |  | *X²* | *df* | *P* value |
| --- | --- | --- | --- | --- |
|  | Time | 177 | 2 | <0.001 |
|  | Location | 249 | 1 | <0.001 |
|  | Time:Location | 40.8 | 2 | <0.001 |
|  |  |  |  |  |
| Mean humidity |  | *X²* | *df* | *P* value |
|  | Time | 59.6 | 2 | <0.001 |
|  | Location | 3.7 | 1 | 0.06 |
|  | Time:Location | 21.8 | 2 | <0.001 |

Models were set up separately.

**Additional file 1: Table S2: Effects of MERF compartments on swarming rate, the height of swarm nucleus and the time at which the swarms started.**

|  | Compartment effects | | |
| --- | --- | --- | --- |
|  | *X²* | *df* | *P* value |
| Swarming rate | 9.8 | 5 | 0.08 |
| Height | 78 | 5 | <0.001 |
| Start time | 69 | 5 | <0.001 |

Models were set up separately.

**Additional file 1: Figure S1.** Light stimuli used into the swarming room. **a.** Ceiling light program with LED panels dimmed from 100% to 0% with the two C_2_ panels turning off 5 minutes after C_1_ panels. **b.** Incandescent bulb light illuminating the bright wall during all the experiment.

**Additional file 1: Figure S2:** Monthly temperatures outside the MERF and in the MERF during experiments.
